# Natural Killer Cell Activity and Response to Neoadjuvant Treatment in Breast Cancer Patients

**DOI:** 10.3390/ijms262110357

**Published:** 2025-10-24

**Authors:** Sofie Høier Gamborg-Kvist, Else Maae, Signe Timm, Ina Mathilde Kjær, Troels Bechmann, Torben Frøstrup Hansen, Line Nederby

**Affiliations:** 1 Department of Oncology, Vejle Hospital, University Hospital of Southern Denmark, 7100 Vejle, Denmark; 2 Institute of Regional Health Research, Faculty of Health Sciences, University of Southern Denmark, 5000 Odense, Denmark; 3 Department of Biochemistry and Immunology, Vejle Hospital, University Hospital of Southern Denmark, 7100 Vejle, Denmark; 4 Department of Oncology, Regional Hospital West Jutland, 7400 Herning, Denmark

**Keywords:** breast cancer, neoadjuvant treatment, natural killer cells, prednisolone, treatment response

## Abstract

No validated biomarkers are available to monitor neoadjuvant treatment effects for breast cancer. Natural killer cell activity (NKA) has shown prognostic potential in other cancers. This study examined the association between NKA and treatment response. Patients had blood samples collected at baseline, before each treatment, and pre- and postoperatively. Plasma IFNγ levels were measured by ELISA as a surrogate marker of NKA, with 250 pg/mL as the cutoff for normal versus low NKA. Study endpoints were residual cancer burden (RCB) class, overall survival (OS), and invasive disease-free survival (IDFS). Seventy-eight patients were included. The five-year IDFS was 88.1% (95% confidence interval (CI) 73.7–94.9%) for patients with normal NKA versus 71.5% (95% CI 40.6–88.2%) for patients with low NKA (*p* = 0.049) preoperatively. At the fifth treatment cycle, the median IFNγ was 11 pg/mL (interquartile range (IQR) 0.5–124 pg/mL) in patients receiving supportive prednisolone and 753 pg/mL (IQR 192–1580 pg/mL) in patients not receiving supportive prednisolone. At the sixth treatment cycle, the corresponding values were 7 pg/mL (IQR 0–15 pg/mL) and 806 pg/mL (IQR 180–1631 pg/mL) (*p* < 0.0001). In conclusion, NKA may have prognostic potential as a biomarker. This study is the first to demonstrate that prednisolone impairs NKA measurement in breast cancer patients.

## 1. Introduction

Breast cancer is the most common type of cancer and the leading cause of cancer death among women worldwide [1]. Neoadjuvant treatment contributes to improved prognosis and minimizes the surgical procedure for high-risk patients intended for curative treatment [2,3,4]. Some patients achieve pathological complete response (pCR) to neoadjuvant treatment that is highly correlated to improved prognosis and survival. Therefore, neoadjuvant treatment response serves as a guide for choosing postoperative treatment strategy [5,6,7]. However, curative treatment can be quite extensive with high risks of side and late effects, and some patients experience no or very little treatment effect and may even experience tumor progression on neoadjuvant therapy. Currently, no known biomarkers are available to monitor the effects of neoadjuvant treatment despite the existing need for predictive and prognostic biomarkers to help stratify patients to the optimal treatment strategy.

Natural killer (NK) cells play an important role in the innate immune system with their ability to kill tumor cells and infected cells without prior sensitization. Moreover, NK cells orchestrate the activation of other immune cells as they secrete cytokines and chemokines that directly or indirectly have anti-tumor and anti-viral effects [8,9,10]. Research has shown a correlation between NK cell activity (NKA) and the risk of developing cancer. Specifically, high NKA was found to be associated with a reduced cancer risk, while low NKA was associated with an increased risk of cancer [11]. This prognostic ability has been replicated in several studies investigating both infiltrating NK cells and NKA in peripheral blood in different cancer types including lung cancer [12,13], colorectal cancer [14] and gastric cancer [15]. Similarly for breast cancer, Dewan and colleagues found NKA to be lower in breast cancer patients as compared to healthy individuals [16]. Later this was confirmed in cohorts of high risk patients with triple negative breast cancer (TNBC) [17,18]. Measurement of NKA can be assessed by utilizing interferon gamma (IFNγ) as a surrogate marker [19,20]. The clinical impact of IFNγ-levels in predicting treatment response has been shown in colorectal cancer [21], prostate cancer [21], ovarian cancer [21], and lung cancer [22]. However, it is well known that the immune response is complex and influenced by several mediators, such as glucocorticoids, which exert inhibitory effects on NK cell functions [23,24,25]. In vitro studies in healthy individuals have demonstrated a similar effect of prednisolone suppressing NKA [26,27,28].

Host immunity generally plays a key role in tumor development. Levels of tumor-infiltrating lymphocytes (TILs) are independent predictors of neoadjuvant treatment response in breast cancer [29,30]. Peripheral blood lymphocytes (PBL), such as NK cells, have been shown to have prognostic value in breast cancer patients treated with neoadjuvant therapy [31], and one study by Feng et al. also showed subsets of PBL, e.g., NK cells, to have a predictive value of treatment response [32]. The clinical value of assessing PBL as opposed to TILs is its availability in simple blood samples at all times, with the potential to be included in a repetitive setting to monitor tumor burden and treatment efficacy.

The purpose of this study was to examine the association between NKA and treatment response and prognosis in patients undergoing neoadjuvant treatment for breast cancer.

## 2. Results

### 2.1. Patient Selection and Characteristics

**Figure 1:**
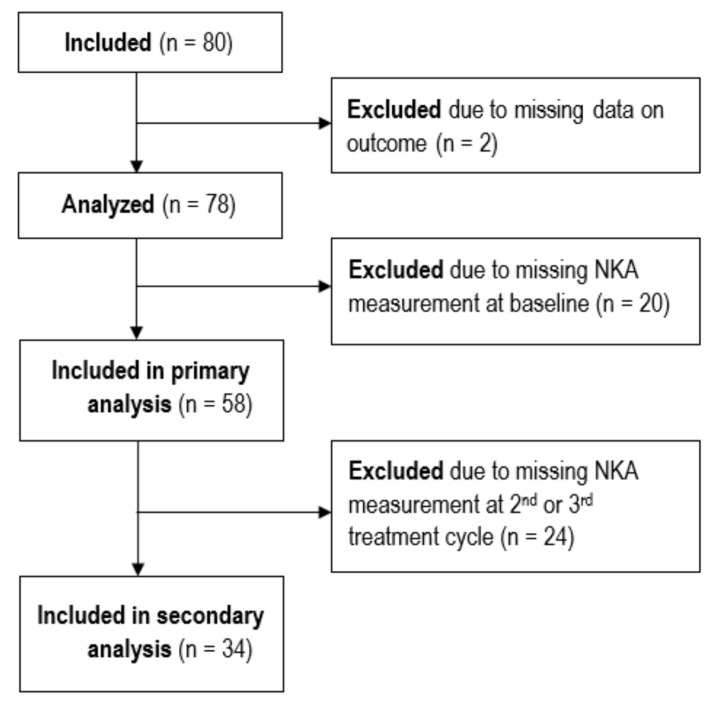
STROBE flow diagram showing the inclusion and exclusion of patients.

Initially, 80 patients met the inclusion criteria. Two patients were diagnosed with metastasis in the sentinel node biopsy prior to neoadjuvant treatment, invalidating the accuracy of the RCB class calculation; hence, they were excluded. A total of 78 patients were thus included in this study, as shown in Figure 1.

Characteristics for all included patients are presented in Table 1 according to baseline NKA. Missing baseline NKA was due either to incorrect handling of the blood sample or to the blood sample being drawn after treatment had been initialized. The cohort showed similar characteristics across all groups.

### 2.2. Association Between NKA and Treatment Response

**Figure 2:**
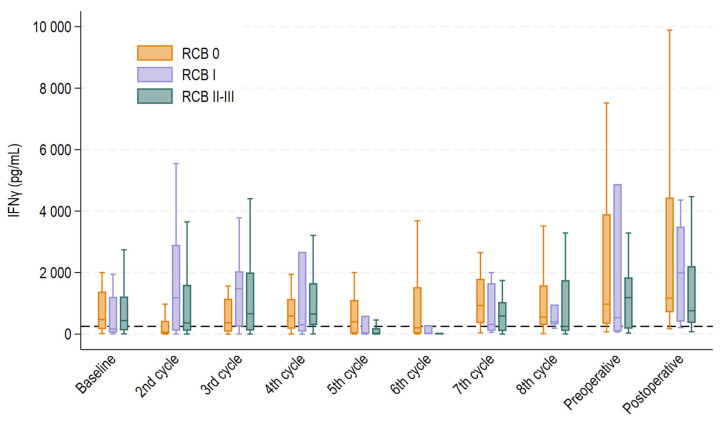
Boxplot displaying median interferon gamma (IFNγ) levels with 25th and 75th percentile at each treatment cycle, pre- and postoperatively for each treatment response group. Whiskers depict the upper and lower adjacent value within 1.5 times the interquartile range. Outliers above the upper adjacent value are not depicted but are included in the statistical analyses.

Levels of IFNγ varied greatly throughout the treatment course and across the RCB groups (Figure 2). The number of patients who had blood samples drawn was consistent across all time points, ensuring that the results were not influenced by variations in sample size. Median IFNγ values at baseline were 472 pg/mL (interquartile range (IQR) 161–1380 pg/mL) for RCB 0; 167 pg/mL (IQR 45–1208 pg/mL) for RCB I; and 439 pg/mL (IQR 122–1219 pg/mL) for RCB II-III (*p* = 0.51). The corresponding medians at midterm (before the fifth treatment cycle) were 400 pg/mL (IQR 21–1107 pg/mL), 16 pg/mL (IQR 14–596 pg/mL), and 11 pg/mL (IQR 4–192 pg/mL) (*p* = 0.01).

We evaluated the association between the baseline NKA and RCB classes using a complete case analysis of the 58 patients with available baseline NKA measurements. A total of 67% of patients responding to neoadjuvant treatment (RCB 0) had normal NKA at baseline (18/27), whereas 50% (3/6) and 64% (16/25) of patients with little or poor response to treatment (RCB I and RCB II/III, respectively) had normal NKA at baseline (*p* = 0.74) (Table 2). Thus, there was no association between baseline NKA and treatment response. Similarly, no associations were found between NKA at baseline and treatment response when using RCB 0/I vs. II/III as an alternative classification and IFNγ at 120 pg/mL as alternative cut-off in sensitivity analyses.

We investigated whether dynamic changes in NKA between samples drawn at baseline, before the second treatment cycle, and before the third treatment cycle were related to response to neoadjuvant treatment. The results are shown in Table 3. Only patients who had a measurement at all three time points were included in this analysis (n = 34). The NKA-high group had persistently normal levels of NKA (IFNγ ≥ 250 pg/mL, n = 10), the NKA-mixed group had a decrease from normal to low levels or vice versa (n = 18), and the NKA-low group had persistently low levels of NKA (IFNγ < 250 pg/mL, n = 6). There was no association between NKA dynamics and response to treatment characterized by RCB class (*p* = 0.67), nor when using alternative RCB class classification or 120 pg/mL as an alternative cut-off in sensitivity analyses.

Stratification on treatment with tocotrienol showed similar results. 

### 2.3. Association Between NKA and Survival Outcomes

We divided the cohort into two groups based on their preoperative NKA. We chose the preoperative sample to avoid the influence of surgical stress response on the immune activity postoperatively. Total risk time was 474.1 person years, with a median follow-up time of 6.3 years (min.: 0.7 years; max.: 7.3 years).

**Figure 3:**
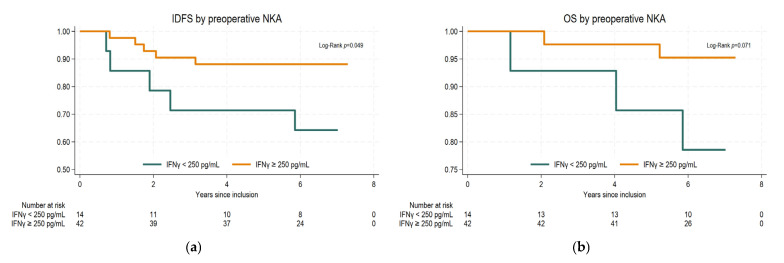
Kaplan–Meier survival curves and log-rank test stratified by preoperative IFNγ levels: (**a**) invasive disease-free survival (IDFS); (**b**) overall survival (OS).

As shown in Figure 3, there was a statistically significant association between preoperative NKA and IDFS. The 5-year IDFS was 88.1% (95% confidence interval (CI): 73.7–94.9%) for patients with normal preoperative NKA and 71.5% (95% CI: 40.6–88.2%) for patients with low preoperative NKA (*p* = 0.049). The 5-year overall survival was 97.6% (95% CI 84.3–99.7%) and 85.7% (95% CI 53.9–96.2%) for normal and low NKA preoperatively, which did not reach statistical significance (*p* = 0.07). Stratification on treatment with tocotrienol showed similar results.

### 2.4. Additional Findings Regarding the Association Between NKA and Treatment with Supportive Prednisolone

Analyses on association between NKA and treatment response were repeated with blood samples not influenced by prednisolone, e.g., patients not treated with prednisolone at the specific time points or patients who were treated with prednisolone but had blood samples drawn prior to prednisolone treatment, specifically on the day before chemotherapy treatment. A total of 50 patients had a baseline NKA measurement not influenced by prednisolone, and only 20 patients had all three consecutive NKA measurements at baseline and before the second and third treatment cycles not influenced by prednisolone. Again, no association was found between either NKA at baseline or dynamic changes in NKA over the first three treatment cycles and treatment responses as characterized by RCB groups (Supplementary Tables S1 and S2).

**Figure 4:**
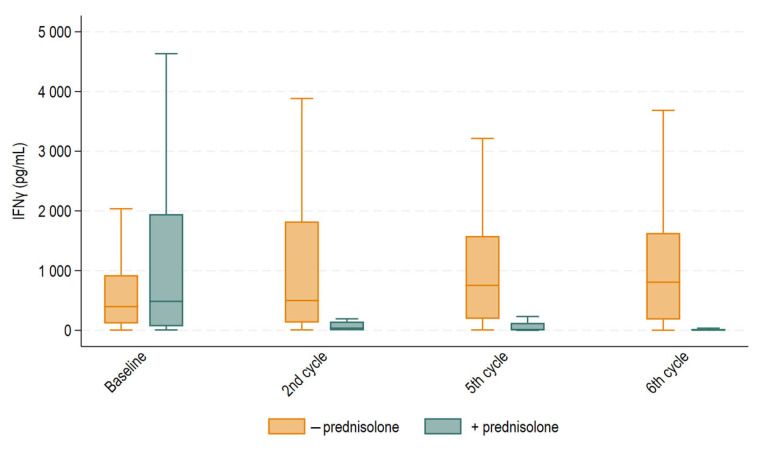
Boxplot displaying median IFNγ levels with 25th and 75th percentile at baseline and before the second, fifth, and sixth treatment cycles for patients treated with supportive prednisolone or not. Whiskers depict the upper and lower adjacent values within 1.5 times the interquartile range. Outliers above the upper adjacent value are not depicted but are included in the statistical analyses. Median IFNγ levels were statistically different before the second, fifth, and sixth treatment cycles (*p* < 0.0001), but not at baseline.

We then compared median IFNγ in patients who received supportive prednisolone prior to treatment with those who did not. Median IFNγ levels for each group are depicted in Figure 4. At baseline (before the first treatment cycle), median IFNγ levels were 485 pg/mL (IQR 67–1946 pg/mL) in patients who received supportive prednisolone and 398 pg/mL (IQR 115–924 pg/mL) in patients who did not (*p* = 0.52). At the second treatment cycle, median IFNγ levels fell to 37 pg/mL (IQR 0–146 pg/mL) for patients who received supportive prednisolone and remained normal at 499 pg/mL (IQR 130–1824 pg/mL) for patients who did not (*p* < 0.0001). At the fifth treatment cycle, median IFNγ levels were 11 pg/mL (interquartile range (IQR) 0.5–124 pg/mL) in patients receiving supportive prednisolone and 753 pg/mL (IQR 192–1580 pg/mL) in patients not receiving supportive prednisolone. At the sixth treatment cycle, the corresponding values were 7 pg/mL (IQR 0–15 pg/mL) and 806 pg/mL (IQR 180–1631 pg/mL) (*p* < 0.0001).

## 3. Discussion

Breast cancer affects a significant number of women each year, but advancements in early diagnosis and more effective systemic therapies have led to improved survival. Consequently, more prevalent side effects and late effects from the extensive curative treatment have emerged. Therefore, there is a need for better prognostic and predictive biomarkers to avoid exposing patients to ineffective treatments.

The present study investigated IFNγ levels (as a surrogate marker for NKA) in patients undergoing neoadjuvant treatment for breast cancer. We examined the association between NKA and treatment response and prognosis, and we presented novel data on the effect of prednisolone to NKA in breast cancer patients.

Our results could not demonstrate an association between NKA at baseline or as dynamic changes over the first three treatment cycles and neoadjuvant treatment response. However, we found a statistically significant better IDFS in patients with a preoperative measurement of normal IFNγ (≥250 pg/mL) as compared to patients with low IFNγ (<250 pg/mL). No difference was found in overall survival.

It was evident that NKA fluctuated greatly across the patient population and over the course of treatment, as shown in Figure 2, which calls for caution in timing measurements and interpreting results. However, levels of IFNγ generally showed a rising tendency towards surgery and even more so for the RCB 0 and RCB I groups, as compared to RCB II-III, potentially indicating better treatment responses in these groups. Figure 2 also shows that levels of IFNγ fell for the RCB 0 group before the second treatment cycle but rose quickly again and remained persistently high (above 250 pg/mL) throughout each treatment cycle. However, before the fifth and sixth treatment cycles, there was a significant decline in levels of IFNγ for both the RCB I and RCB II-III groups relative to the RCB 0 group. This supports previous findings that lower NKA is associated with an increased cancer burden [16] and aligns with a study by Verma et al. examining the association between NKA and response to neoadjuvant breast cancer treatment. They found that patients who achieved pCR had higher levels of NKA after neoadjuvant treatment [34]. In 2024, Kim and colleagues proved that higher rates of specific subtypes of NK cells in peripheral blood after neoadjuvant treatment were associated with a better treatment response, indicating that a robust NK response aids in better tumor eradication [35].

While evaluating the findings presented in Figure 2, we contemplated the marked reduction in NKA observed at the fifth and sixth treatment cycles. By investigating the treatment courses, we realized that the observed effect might be explained by administration of supportive prednisolone. Prednisolone as hypersensitivity prophylaxis was administered either prior to the first and second or fifth and sixth treatment cycles in conjunction with taxane-based chemotherapy. All patients in the cohort were exposed to prednisolone as hypersensitivity prophylaxis at some point in the treatment course. IFNγ levels were significantly lower in patients treated with supportive prednisolone immediately prior to blood samples drawn before the second, fifth and sixth treatment compared to those who were not. Blood samples for the first treatment cycle (baseline) were often drawn in relation to patients’ first consultation before administration of prednisolone, which likely explains why the same suppressive effect was not observed at that time point. Our results align with those of previous studies reporting the suppressive ability of prednisolone on NKA in in vitro environments [26,27]. However, to our knowledge, our study is the first to report this suppressive effect in humans through this approach.

The immunomodulatory effects of prednisolone are well-known and utilized in various cancer treatments. Within breast cancer treatment, prednisolone is used to manage side effects, such as nausea, and to improve appetite and quality of life. Moreover, it is used to prevent allergic reactions to taxane-based chemotherapy for the first two treatments when hypersensitivity reactions are most common. If no such reactions occur, supportive prednisolone is typically discontinued. However, the negative impact of prednisolone on NKA may impair the cytotoxic ability of NK cells against tumor cells, potentially reducing the overall treatment response. To date, no studies have directly evaluated the impact of the use of prednisolone on treatment response in breast cancer treatment. It comes as no surprise that prednisolone elicits this immunosuppressive effect to NKA, though it raises several interesting questions. Although the molecular mechanisms of NK cells in anti-tumor activities are not fully clarified, it is well known that NK cells execute very important roles in cancer control both directly and indirectly as elaborated in several review articles [8,9,10,36]. For instance, studies have shown that higher rates of NK-cells in the tumor microenvironment were associated with better clinical outcomes. Liu and colleagues conducted a study on patients with TNBC, and by dividing the patients into high- and low-risk groups based on a risk score related to NK-cell related genes, tumor immune cells were found to be highly infiltrated in the low risk groups, who were also proposed to have better immunotherapeutic response [17]. In another TNBC cohort, Jin et al. found that the presence of NK cells in the tumor microenvironment inhibits the further invasion of breast cancer cells and was related to a better OS [37]. Mounting evidence marks the importance of NK cells in battling cancer, and taken together with the findings of our study, it is noteworthy that administration of supportive prednisolone impairs the immune reaction as such. It remains unclear whether the effect of prednisolone is short-lived or sustained over a longer period; however, evaluation of NKA in prednisolone-affected samples is compromised and should be interpreted with caution. Furthermore, our findings raise the hypothesis that an altered premedication schedule, dose reduction, or earlier administration of supportive prednisolone on the day of treatment could potentially minimize the impairment on NKA, which may contribute to a stronger immune response, though this remains to be validated. In fact, a study by Quock et al. has shown that treatment with dexamethasone/prednisolone can safely be omitted after the first treatment with taxane-based chemotherapy without increasing the risk of subsequent hypersensitivity reactions [38]. To elaborate on this, further studies examining the in vitro and in vivo dose–response relationship of prednisolone to NKA are warranted.

This study was strengthened by its longitudinal design, with multiple NKA measurements throughout the course of the neoadjuvant treatment schedule, as well as pre- and postoperatively. However, the results must be interpreted with caution, as IFNγ levels at the fifth and sixth treatment cycles appeared to be strongly influenced by prednisolone. The risk of bias related to the descriptive observation of NKA patterns is considered low since prednisolone was administered irrespective of treatment response and prior to its assessment. Moreover, subgroup analyses of blood samples not affected by prednisolone did not alter the primary findings.

A probable limitation, however, is confounding by indication, as HER2-positive tumors often respond very well to neoadjuvant treatment and may therefore be overrepresented in the RCB 0 group. HER2-positive patients did not receive supportive prednisolone before blood samples drawn at the fifth and sixth cycles due to treatment regimen, suggesting that the observed differences in NKA between response groups at the fifth and sixth treatment cycles could reflect a regimen effect rather than a true association between NKA and treatment response. Additionally, all patients were treated with a reduced dose of prednisolone for antiemetic purposes on the same day and the three following days after epirubicin and cyclophosphamide. This study did not account for that effect, as prednisolone as antiemetic treatment was not administered until after blood sampling in each treatment cycle. Therefore, we cannot rule out that NKA was affected to some extent during all treatment cycles as prednisolone was administered either for antiallergic or antiemetic purposes. The prognostic analyses remain valid as the preoperative samples were not influenced by prednisolone since the drug was not administered at this stage—neither for antiallergic, antiemetic, nor pre-surgical indications.

It must be taken into consideration that breast cancer is a very heterogenous disease, which is even more evident in a small cohort. Hence, this study is limited by its low statistical power. It included 78 patients, but complete case analyses were only possible for 58 and 34 patients (Table 2 and Table 3), with even fewer in the subgroup analyses. This was due to challenges with blood sampling setup and laboratory processing, which affected the completion of all planned NKA analyses; hence, we assume missing data to be missing completely at random. Furthermore, the original phase II trial was designed to assess the frequency of pCR in a trial with patients randomized to treatment with tocotrienol. Although the trial was also intended for analysis of NKA, the power calculation did not account for the objective of this study; therefore, this study is likely underpowered and at increased risk of type II error. Only seven deaths were reported in this cohort, and another 12 patients experienced relapses. This precludes robust multivariable Cox regression, as including several prognostic factors with so few events would risk severe model overfitting and unstable estimates. Therefore, we present unadjusted survival analyses, and the findings should be interpreted with caution. Future studies with larger cohorts are needed to clarify the independent prognostic impact of NKA.

In conclusion, this study found that patients with normal preoperative NKA had significantly longer IDFS, indicating that NKA may have potential as a prognostic biomarker. Patients obtaining complete response to neoadjuvant treatment generally had higher levels of NKA, although these results must be interpreted with great caution due to the effects of prednisolone and probable confounding of HER2 status. Nonetheless, the consistent trends observed over time indicate that NKA may serve as a relevant biomarker for treatment response and prognosis, although careful consideration of measurement timing is warranted. Supportive prednisolone significantly affected NKA, compromising prednisolone-affected NKA measurements and potentially impairing immune function. To ensure valid evaluation of NKA as a prognostic and predictive biomarker, we suggest that blood samples should be collected prior to prednisolone administration. Further studies exploring the dose–response relationship of prednisolone to NKA as well, as the predictive and prognostic value of NKA in larger cohorts, are warranted.

## 4. Materials and Methods

### 4.1. Study Design and Study Population

The study population originates from a randomized phase II trial conducted at the Department of Oncology, Vejle Hospital, Denmark, from 2016 to 2019, investigating the clinical effects of supportive vitamin E treatment to neoadjuvant treatment of breast cancer (ClinicalTrials.gov NCT02909751 (registered on 16 September 2016), Regional Committee on Health Research Ethics for Southern Denmark S-20160009 (approved on 7 March 2016), Danish Medical Agency 2,016,034,241 (approved on 21 July 2016)). The trial included women with newly diagnosed breast cancer eligible for neoadjuvant treatment according to prevailing guidelines. Inclusion criteria were histologically verified adenocarcinoma of the breast, age above 18 years, Eastern Cooperative Oncology Group performance status 0–2, and normal organ functions. Eligibility criteria can be found in the article by Kjær et al. [33]. The patients were randomized to either standard neoadjuvant treatment or in combination with tocotrienol (vitamin E), which studies have indicated has antineoplastic effects without harmful effects on normal cells [39]. Primary results from the trial showed no difference in neoadjuvant treatment response, as evaluated by residual cancer burden (RCB) class between the randomization groups [33]. Hence, the randomization groups were pooled into one observational cohort for the present study. All patients gave written and orally informed consent to participate.

### 4.2. Treatment and Follow-Up

All patients received neoadjuvant treatment according to national guidelines by the Danish Breast Cancer Group (DBCG). Patients with HER2-negative breast cancer were treated with four cycles of epirubicin and cyclophosphamide (EC) every three weeks followed by four cycles of docetaxel every three weeks or weekly paclitaxel. Patients with HER2-positive breast cancer were treated with four cycles of docetaxel or paclitaxel concomitant with trastuzumab alone or in combination with pertuzumab followed by four cycles of EC.

Administration of supportive prednisolone for hypersensitivity prophylaxis was registered. If not otherwise specified in patient records, we assumed that prednisolone was administered according to prevailing guidelines, i.e., 100 mg prednisolone 4–6 h prior to the first two cycles of taxane-based chemotherapy (cycles 1–2 for patients with HER2-positive tumors and cycles 5–6 for patients with HER2-negative tumors). Prednisolone, as antiemetic prophylaxis, was not registered as it would not be administered until after blood sampling.

Patients randomized to treatment with tocotrienol received additional oral tocotrienol three times a day from the first day of chemotherapy till surgery. Patients randomized to standard treatment received placebo. Patients then underwent surgery with removal of tumor and axillary lymph nodes according to national guidelines from DBCG. For further details, we refer to the clinical study by Kjær et al. [33].

Treatment response was evaluated by sequential MRI scans during neoadjuvant treatment and with assessment of the pathological treatment response in the surgical specimen based on the Miller–Payne grading. This was converted to residual cancer burden (RCB) class on a scale from pathological complete response (pCR), i.e., RCB 0, to significant tumor burden, i.e., RCB III [40].

Study endpoints were overall survival (OS) and invasive disease-free survival (IDFS), defined as the time from primary surgery to one of the following events: ipsilateral invasive breast cancer, regional breast cancer recurrence, contralateral invasive breast cancer, distant recurrence, death of any cause, or second non-breast invasive cancer, excluding all in situ cancers, squamous, and basal skin cancers. Patients were censored on 31 December 2023.

### 4.3. Blood Samples, Laboratory Methods, and NKA Measurement

**Figure 5:**
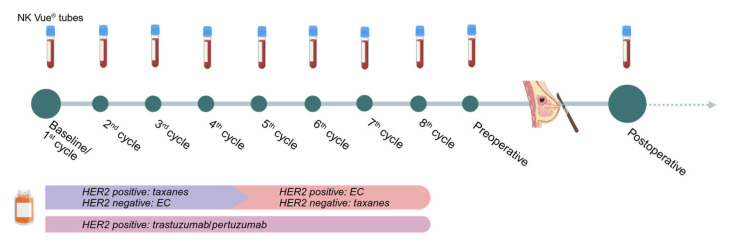
Overview of treatment schedule and blood sample collection. The timeline illustrates the concurrence between neoadjuvant chemotherapy and surgery in relation to treatment cycles and blood samples. EC: epirubicin/cyclophosphamide; taxanes: taxane-based chemotherapy, e.g., paclitaxel or docetaxel. Created in https://BioRender.com, accessed on 3 September 2025.

Blood samples for NKA measurement were drawn according to Figure 5, e.g., at baseline before commencing neoadjuvant treatment, before each treatment cycle, and before and after surgery. Blood samples were either drawn the day before or on the day of treatment approx. 1–2 h prior to treatment start. Since prednisolone was administered between 4 and 6 h before treatment, blood samples were drawn approx. 2–5 h after prednisolone intake. A total of 1 mL of whole blood was drawn into NK Vue^®^ tubes (NKMAX, Seongnam-si, Republic of Korea) and placed in an incubator of 37 °C within 15 min. After 20–24 h, plasma was collected and stored at −80 °C for a maximum of one month. Levels of IFNγ were quantified in plasma using the NK Vue^®^ enzyme-linked immunosorbent assay (ELISA) (NKMAX) according to the manufacturer’s instructions. In brief, plasma samples were thawed and centrifuged for 1 min at 11,600× *g* before being added into the pre-coated ELISA wells—both directly and as a 1:10 dilution. A standard curve dilution (62.5–2000 pg/mL), two controls provided in the kit, and three additional control serum samples (200 pg/mL, 1500 pg/mL, and 3600 pg/mL) were included on each plate in duplicates. After 1 h of incubation, the plates were thoroughly washed and then incubated for 1 h 30 min with anti-IFNγ, with horseradish peroxidase attached via biotin–streptavidin interaction. Plates were washed again and incubated with tetramethyl benzidine substrate solution. After 30 min, stop solution was added, and absorbance was immediately measured at 450 nm. The in-house intra-assay coefficient of variation (CV) was <10%, and the inter-assay CV was <12%. According to the manufacturer’s recommendations, a cut-off of 250 pg/mL was applied to separate a normal from an abnormal NKA sample. Measured in-house, the lower reference limit was 120 pg/mL.

### 4.4. Statistics

Descriptive statistics were used to summarize the demographic and clinical characteristics of the study population. Categorical variables were presented as frequencies and percentages, while continuous variables were reported as mean and min–max range. Kruskal–Wallis test was used to compare levels of IFNγ between RCB groups. We used chi-squared test to analyze the association between normal vs. low NKA and RCB class grouped as RCB 0 vs. RCB I vs. RCB II/III. All time-to-event endpoints were analyzed using Kaplan–Meier survival statistics with Log-rank tests.

Stratification was performed based on treatment with tocotrienol. Sensitivity analyses based on a cut-off of 120 pg/mL and altered RCB class division (RCB 0/I vs. RCB III/III) were conducted to assess the robustness of the findings. In addition, we performed subgroup analyses of the association between NKA and RCB class using blood samples not influenced by prednisolone intake to avoid potential corticosteroid-induced suppression of NKA.

All statistical analyses were performed using STATA BE18 (StataCorp LLC, College Station, TX, USA). A *p*-value of less than 0.05 was considered statistically significant.

## Figures and Tables

**Table 1:** Descriptive characteristics of participants presented as frequencies (%) or mean (min–max).

	Normal NKA at Baseline (n = 37)	Low NKA at Baseline (n = 21)	Missing Baseline NKA Measurement (n = 20)	All (n = 78)
Age	55.7 (28.5–82.8)	56.0 (33.9–79.8)	52.4 (36.4–77.0)	55.0 (28.5–82.8)
Menopausal status				
Pre	17 (46%)	10 (48%)	11 (55%)	38 (49%)
Post	20 (54%)	11 (52%)	9 (45%)	40 (51%)
Performance status				
0	36 (97%)	18 (86%)	20 (100%)	74 (95%)
1	-	2 (9%)	-	2 (3%)
Missing	1 (3%)	1 (5%)	-	2 (2%)
ER status (pre-treatment biopsy) ^a^				
Positive (≥1%)	25 (68%)	13 (62%)	14 (70%)	52 (67%)
Negative (0%)	12 (32%)	8 (38%)	6 (30%)	26 (33%)
HER2 status (pre-treatment biopsy) ^b^				
Positive	14 (38%)	9 (43%)	7 (35%)	30 (38%)
Negative	23 (62%)	12 (57%)	13 (65%)	48 (62%)
Histological type (combined biopsy and surgical specimen)				
Ductal	24 (65%)	12 (57%)	13 (65%)	49 (63%)
Lobular	3 (8%)	-	-	3 (4%)
Other/unknown	10 (27%)	9 (43%)	7 (35%)	26 (33%)
Tumor size (baseline MRI)				
T1 < 20 mm	6 (16%)	2 (10%)	1 (5%)	9 (12%)
T2 > 21 mm < 50 mm	17 (46%)	15 (71%)	12 (60%)	44 (56%)
T3 > 50 mm	11 (30%)	2 (10%)	7 (35%)	20 (26%)
MRI not performed	3 (8%)	2 (9%)	-	5 (6%)
Pathological lymph nodes ^c^				
Yes	23 (62%)	14 (67%)	15 (75%)	52 (67%)
No	14 (38%)	7 (33%)	5 (25%)	26 (33%)
Malignancy grade (pre-treatment biopsy)				
1	-	1 (5%)	-	1 (1%)
2	16 (43%)	6 (29%)	8 (40%)	30 (39%)
3	8 (22%)	6 (29%)	5 (25%)	19 (25%)
Unknown	13 (35%)	8 (37%)	7 (35%)	28 (35%)
Treatment with tocotrienol ^d^				
Yes	9 (24%)	13 (62%)	14 (70%)	36 (46%)
No	28 (73%)	8 (38%)	6 (30%)	42 (53%)

^a^ Estrogen receptor status. ^b^ Status of human epidermal growth factor receptor 2 (HER2) in breast cancer tumor evaluated by immunohistochemistry (IHC) and fluorescence in situ hybridization (FISH). Positive: IHC 3+ or IHC 2+ and FISH >2. Negative: IHC 0 or IHC 1+ or IHC 2+ and FISH <2. ^c^ Malignant cells in lymph node biopsy, malignant cells in sentinel lymph node preoperatively, or malignant cells or treatment response in lymph node removed at time of breast cancer surgery. ^d^ See clinical study by Kjær et al. [33].

**Table 2:** Association between baseline NKA and RCB class.

Baseline NK Cell Activity	RCB Class			
	0	I	II + III	Total
IFNγ < 250 pg/mL	9 (33%)	3 (50%)	9 (36%)	21
IFNγ ≥ 250 pg/mL	18 (67%)	3 (50%)	16 (64%)	37
Total	27	6	25	58

A 2 × 3 contingency table for the chi-squared test of the association between levels of IFNγ at baseline and responses to neoadjuvant treatment characterized as the residual cancer burden (RCB) class. Results are shown as frequencies (%). *p* = 0.74.

**Table 3:** Association between NKA dynamics and RCB class.

Dynamics in NK Cell Activity from Baseline to 3rd Treatment Cycle	RCB Class			
	0	I	II + III	Total
NKA-high (all IFNγ ≥ 250 pg/mL)	3	2	5	10
NKA-low (all IFNγ < 250 pg/mL)	4	1	1	6
NKA-mixed (varying IFNγ levels)	9	2	7	18
Total	16	5	13	34

A 3 × 3 contingency table for the chi-squared test of the association between dynamic changes in NKA over the first three treatments and responses to neoadjuvant treatment characterized as the residual cancer burden (RCB) class. Results are shown as frequencies (%). *p* = 0.67.

## Data Availability

Due to Danish data protection regulations and patient confidentiality, the datasets are not publicly available. Access to anonymized data may be granted upon reasonable request and with appropriate permissions from the relevant institutional and ethical review boards.

## Supplementary Materials

The following supporting information can be downloaded at https://www.mdpi.com/article/10.3390/ijms262110357/s1.

## Abbreviations

The following abbreviations are used in this manuscript:

IDFS	Invasive disease free survival
IFN*γ*	Interferon-gamma
NKA	Natural killer cell activity
OS	Overall survival
PBL	Peripheral blood lymphocytes
RCB	Residual cancer burden
TILs	Tumor infiltrating lymphocytes
TNBC	Triple negative breast cancer
