# Supplementary material for: Natural Killer Cell Activity and Response to Neoadjuvant Treatment in Breast Cancer Patients
Source: Int J Mol Sci. 2025 Oct 24;26(21):10357. doi: 10.3390/ijms262110357 (PMC12609923; doi:10.3390/ijms262110357)
Supplement: Supplementary file 1 [file ijms-26-10357-s001.zip › ijms-3898858-supplementary.pdf]

Supplementary Table S1. Association between baseline NKA and RCB class for non-prednisolone affected blood samples.

Baseline NK cell activity	RCB class			
	0	I	II + III	Total
IFNγ < 250 pg/mL	6	3	9	18
IFNγ ≥ 250 pg/mL	14	3	15	32
Total	20	6	24	50

2x3 contingency table with chi-squared test of the association between levels of IFNγ at baseline and response to neoadjuvant treatment characterized as residual cancer burden (RCB) class for blood samples not influenced by prednisolone. Results shown as frequencies (%). p = 0.66.

Supplementary Table S2. Association between NKA dynamics and RCB class for non-prednisolone affected blood samples.

Dynamics in NK cell activity from baseline to 3rd treatment cycle	RCB class			
	0	I	II + III	Total
NKA-high (all IFNγ ≥ 250 pg/mL)	1	2	5	8
NKA-low (all IFNγ < 250 pg/mL)	1	0	1	2
NKA-mixed (varying IFNγ levels)	3	1	6	10
Total	5	3	12	20

3x3 contingency table with chi-squared test of the association between dynamic changes in NKA over the first three treatments and response to neoadjuvant treatment characterized as residual cancer burden (RCB) class for blood samples not influenced by prednisolone. Results shown as frequencies (%). p = 0.71.
